# 2-(4-Chloro­phen­oxy)acetohydrazide

**DOI:** 10.1107/S1600536809049538

**Published:** 2009-11-25

**Authors:** Grzegorz Dutkiewicz, C. S. Chidan Kumar, B. Narayana, H. S. Yathirajan, Maciej Kubicki

**Affiliations:** a Department of Chemistry, Adam Mickiewicz University, Grunwaldzka 6, 60-780 Poznań, Poland; b Department of Studies in Chemistry, University of Mysore, Manasagangotri, Mysore 570 006, India; c Department of Studies in Chemistry, Mangalore University, Mangalagangotri 574 199, India

## Abstract

In the title compound, C_8_H_9_ClN_2_O_2_, the two planar fragments, *i.e.* the chloro­phenyl and C—C(=O)—N groups, are inclined at 14.93 (17)°. In the crystal, relatively weak inter­molecular N—H⋯N, C—H⋯O and N—H⋯O hydrogen bonds connect the mol­ecules into layers. The hydro­phobic parts of mol­ecules stick outside these layers and are connected with the neighbouring layers only by van der Waals contacts and Cl⋯Cl inter­actions [3.406 (2) Å].

## Related literature

For background to hydrazides, see: Cajocorius *et al.* (1977 ▶); Liu *et al.* (2006 ▶); Narayana *et al.* (2005 ▶). For related structures, see: Akhtar *et al.* (2009 ▶); Lokanath *et al.* (1998 ▶); Mahendra *et al.* (2004 ▶); Podyachev *et al.* (2007 ▶). For graph-set symbols, see: Bernstein *et al.* (1995 ▶). For halogen–halogen inter­actions, see: Pedireddi *et al.* (1994 ▶).
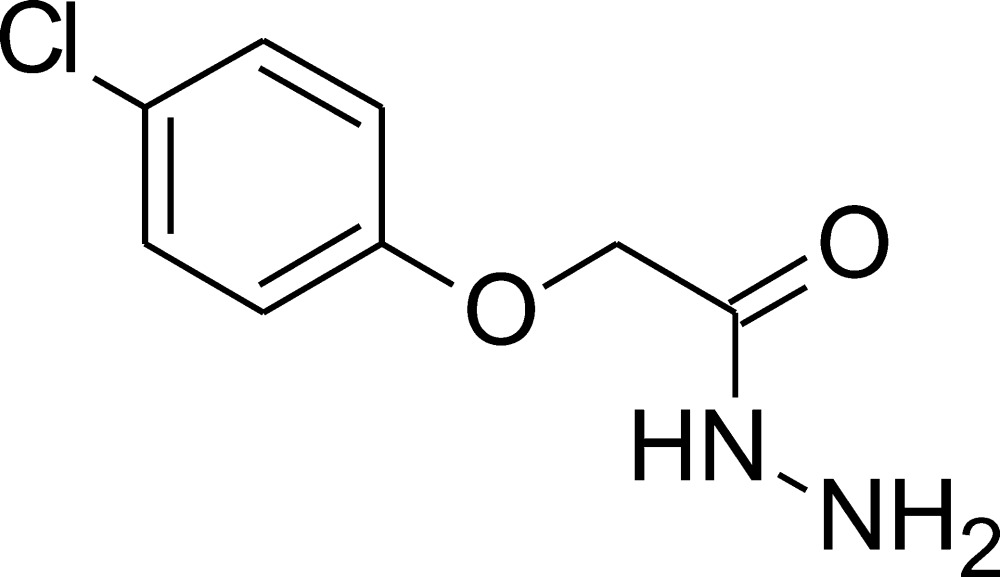


## Experimental

### 

#### Crystal data

C_8_H_9_ClN_2_O_2_
*M* *_r_* = 200.62
Monoclinic, 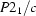

*a* = 6.444 (1) Å
*b* = 4.011 (1) Å
*c* = 35.369 (4) Å
β = 91.89 (1)°
*V* = 913.7 (3) Å^3^
*Z* = 4
Mo *K*α radiation
μ = 0.39 mm^−1^
*T* = 295 K
0.4 × 0.4 × 0.15 mm

#### Data collection

Oxford Diffraction Xcalibur (Sapphire2, large Be window) diffractometer
Absorption correction: multi-scan (*CrysAlis Pro*; Oxford Diffraction, 2009 ▶) *T*_min_ = 0.678, *T*_max_ = 0.944
2864 measured reflections
1761 independent reflections
1448 reflections with *I* > 2σ(*I*)
*R*_int_ = 0.022

#### Refinement

*R*[*F* ^2^ > 2σ(*F* ^2^)] = 0.058
*wR*(*F* ^2^) = 0.118
*S* = 1.15
1761 reflections
154 parameters
All H-atom parameters refined
Δρ_max_ = 0.22 e Å^−3^
Δρ_min_ = −0.28 e Å^−3^

### 

Data collection: *CrysAlis Pro* (Oxford Diffraction, 2009 ▶); cell refinement: *CrysAlis Pro*; data reduction: *CrysAlis Pro*; program(s) used to solve structure: *SIR92* (Altomare *et al.*, 1993 ▶); program(s) used to refine structure: *SHELXL97* (Sheldrick, 2008 ▶); molecular graphics: *Stereochemical Workstation Operation Manual* (Siemens, 1989 ▶); software used to prepare material for publication: *SHELXL97*.

## Supplementary Material

Crystal structure: contains datablocks I, global. DOI: 10.1107/S1600536809049538/is2496sup1.cif

Structure factors: contains datablocks I. DOI: 10.1107/S1600536809049538/is2496Isup2.hkl

Additional supplementary materials:  crystallographic information; 3D view; checkCIF report

## Figures and Tables

**Table 1:** Hydrogen-bond geometry (Å, °)

*D*—H⋯*A*	*D*—H	H⋯*A*	*D*⋯*A*	*D*—H⋯*A*
N1—H1*A*⋯O4^i^	0.93 (4)	2.51 (4)	3.160 (3)	127 (3)
N1—H1*B*⋯O4^ii^	0.89 (4)	2.15 (4)	3.020 (3)	165 (3)
N2—H2⋯N1^iii^	0.86 (3)	2.23 (3)	2.997 (3)	149 (3)
C8—H8⋯O4^iv^	0.94 (3)	2.51 (3)	3.376 (3)	153 (2)

Symmetry codes: (i) 
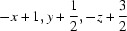
; (ii) 
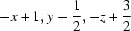
; (iii) 
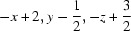
; (iv) 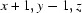.

**Fig. 1.**
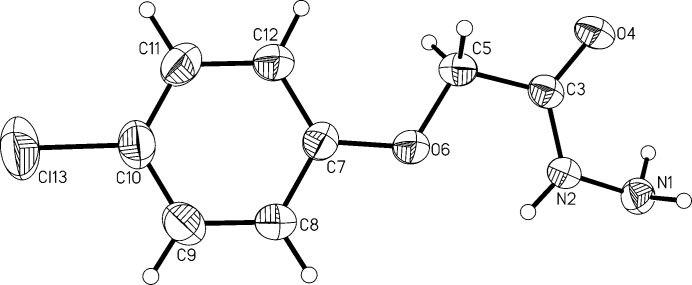
Anisotropic ellipsoid representation of the compound I together with atom labelling scheme. The ellipsoids are drawn at the 50% probability level and hydrogen atoms are depicted as spheres with arbitrary radii.

**Fig. 2.**
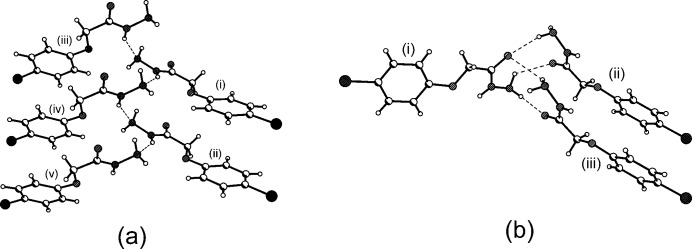
The hydrogen bonded motifs in the crystal structure of I. Hydrogen bonds are shown as dashed lines. (a) the N—H···N chain. [Symmetry codes: (i) x, y, z; (ii) x, -3/2 + y, 3/2 - z; (iii) 2 - x, 1/2 + y, 3/2 - z; (iv) 2 - x, -1/2 + y, 3/2 - z; (v) 2 - x, -3/2 + y, 3/2 - z.] (b) the N—H···O chains and rings. [Symmetry codes: (i) x, y, z; (ii) 1 - x, 1/2 + y, 3/2 - z; (iii) 1 - x, -1/2 + y, 3/2 - z.]

**Fig. 3.**
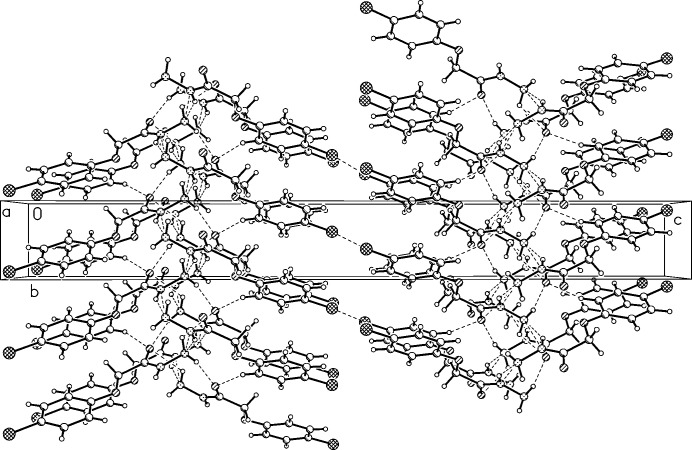
Crystal packing as seen along the a axis. Hydrogen bonds and Cl···Cl contacts are shown as dashed lines.

## supplementary crystallographic information

### Comment

Hydrazides are useful precursors in the synthesis of several heterocyclic systems (*e.g.*, Narayana *et al.*, 2005). Some substituted hydrazides are reported to exhibit carcinostatic activity against several types of tumors and also possess antimicrobial activity (*e.g.*, Cajocorius *et al.*, 1977). They are also used as intermediates in many pharmaceutically important compounds (Liu *et al.*, 2006). A new hydrazide, 2-(4-chlorophenoxy)acetohydrazide (I, Scheme 1), C_8_H_9_ClN_2_O_2_ was synthesized and its crystal structure is reported.

The molecule of I consists of two planar fragments (Fig. 1): the phenyl ring [maximum deviation of 0.014 (2) Å] and the N—C(=O)—C group, which is planar within 0.008 (2) Å. The N1 and O6 atoms deviate significantly (by *ca* 0.11 Å), and in the opposite directions, from this latter plane. Overall, the molecule is only slightly bent as the dihedral angle between the planes described above is 14.93 (17)°. Even smaller values of this angle were observed in similar compounds: 5.0° in [2-methyl-4-(2-methylbenzoyl)-phenoxy]acetohydrazide (Mahendra *et al.*, 2004), 3.6° in (2,4-dichlorophenoxy)acetohydrazide (Lokanath *et al.*, 1998) or 5.7° in 4-*tert*-butylphenoxyacetohydrazide (Podyachev *et al.*, 2007). This planar and (*Z*)-NCCO conformation was sometimes ascribed to the doubtful intramolecular N—H···O hydrogen bond. When the steric hindrance is present, as for instance in the structure of 2-(4-bromophenoxy)propanohydrazide (Akhtar *et al.*, 2009), the two planar fragments become almost perpendicular, dihedral angle between them is 84.9°.

In the crystal structure rather long intermolecular hydrogen bonds connect molecules into three-dimensional network (Table 1). The N—H···N hydrogen bonds, for which the terminal nitrogen atom of NH_2_ group acts as an acceptor, make a C(3) graph-set motif (Bernstein *et al.*, 1995) - the chain of molecules along the *b* axis. Two N—H···O hydrogen bonds between the NH_2_ group and carbonyl oxygen atoms from neighbouring molecules make antiparallel C(5) chains that are interwoven into subsequent *R*^2^_2_(10) rings. In the crystal structure there are layers of molecules connected by these hydrogen bonded hydrophilic fragments and the hydrophobic chlorophenyl fragments stick outside the layers. There are relatively short and linear Cl···Cl contacts between these layers [Cl13···Cl13(3 - *x*,-1 - *y*,1 - *z*) 3.406 (2) Å, C10—Cl13···Cl13(3 - *x*,-1 - *y*,1 - *z*) 155.14 (13)°], suggesting that there is a possibility for "dihalogen" interactions (*e.g.* Pedireddi *et al.*, 1994).

### Experimental

A mixture of ethyl(4-chlorophenoxy)acetate (21.4 g, 0.1 mol) and 6.0 ml of hydrazine hydrate in 90 ml of ethanol was refluxed over water bath for 6 h. The precipitate formed was filtered and recrystallized from ethanol (m.p.: 425 K). Analysis for C_8_H_9_ClN_2_O_2_: Found (Calculated): C 47.89 (47.81), H 4.52 (4.48), N 13.96% (13.88%).

### Refinement

All hydrogen atoms were freely refined.

### Crystal data

**Table:**

C_8_H_9_ClN_2_O_2_	*F*(000) = 416
*M**_r_* = 200.62	*D*_x_ = 1.458 Mg m^−^^3^
Monoclinic, *P*2_1_/*c*	Mo *K*α radiation, λ = 0.71073 Å
Hall symbol: -P 2ybc	Cell parameters from 1266 reflections
*a* = 6.444 (1) Å	θ = 2.3–26.8°
*b* = 4.011 (1) Å	µ = 0.39 mm^−^^1^
*c* = 35.369 (4) Å	*T* = 295 K
β = 91.89 (1)°	Plate, colourless
*V* = 913.7 (3) Å^3^	0.4 × 0.4 × 0.15 mm
*Z* = 4	

### Data collection

**Table:**

Oxford Diffraction Xcalibur (Sapphire2, large Be window) diffractometer	1761 independent reflections
Radiation source: Enhance (Mo) X-ray Source	1448 reflections with *I* > 2σ(*I*)
graphite	*R*_int_ = 0.022
Detector resolution: 8.1929 pixels mm^-1^	θ_max_ = 26.8°, θ_min_ = 2.3°
ω–scan	*h* = −8→6
Absorption correction: multi-scan (*CrysAlis PRO*; Oxford Diffraction, 2009)	*k* = −3→4
*T*_min_ = 0.678, *T*_max_ = 0.944	*l* = −31→43
2864 measured reflections	

### Refinement

**Table:**

Refinement on *F*^2^	Primary atom site location: structure-invariant direct methods
Least-squares matrix: full	Secondary atom site location: difference Fourier map
*R*[*F*^2^ > 2σ(*F*^2^)] = 0.058	Hydrogen site location: difference Fourier map
*wR*(*F*^2^) = 0.118	All H-atom parameters refined
*S* = 1.15	*w* = 1/[σ^2^(*F*_o_^2^) + (0.0271*P*)^2^ + 1.1206*P*] where *P* = (*F*_o_^2^ + 2*F*_c_^2^)/3
1761 reflections	(Δ/σ)_max_ = 0.002
154 parameters	Δρ_max_ = 0.22 e Å^−^^3^
0 restraints	Δρ_min_ = −0.28 e Å^−^^3^

### Special details

**Table:**

Geometry. All e.s.d.'s (except the e.s.d. in the dihedral angle between two l.s. planes) are estimated using the full covariance matrix. The cell e.s.d.'s are taken into account individually in the estimation of e.s.d.'s in distances, angles and torsion angles; correlations between e.s.d.'s in cell parameters are only used when they are defined by crystal symmetry. An approximate (isotropic) treatment of cell e.s.d.'s is used for estimating e.s.d.'s involving l.s. planes.
Refinement. Refinement of *F*^2^ against ALL reflections. The weighted *R*-factor *wR* and goodness of fit *S* are based on *F*^2^, conventional *R*-factors *R* are based on *F*, with *F* set to zero for negative *F*^2^. The threshold expression of *F*^2^ > σ(*F*^2^) is used only for calculating *R*-factors(gt) *etc*. and is not relevant to the choice of reflections for refinement. *R*-factors based on *F*^2^ are statistically about twice as large as those based on *F*, and *R*- factors based on ALL data will be even larger.

### Fractional atomic coordinates and isotropic or equivalent isotropic displacement parameters (Å^2^)

**Table:**

	*x*	*y*	*z*	*U*_iso_*/*U*_eq_	
N1	0.7883 (4)	0.5362 (7)	0.75916 (7)	0.0346 (6)	
H1A	0.717 (5)	0.733 (11)	0.7541 (10)	0.066 (11)*	
H1B	0.711 (5)	0.417 (9)	0.7747 (9)	0.054 (10)*	
N2	0.8013 (3)	0.3650 (6)	0.72417 (6)	0.0317 (5)	
H2	0.909 (4)	0.247 (8)	0.7201 (8)	0.039 (8)*	
C3	0.6568 (4)	0.4000 (7)	0.69716 (7)	0.0300 (6)	
O4	0.4957 (3)	0.5630 (6)	0.70086 (5)	0.0425 (5)	
C5	0.6937 (4)	0.2401 (8)	0.65965 (8)	0.0341 (6)	
H5A	0.573 (5)	0.103 (8)	0.6525 (8)	0.047 (9)*	
H5B	0.709 (4)	0.412 (8)	0.6420 (8)	0.043 (8)*	
O6	0.8781 (3)	0.0467 (5)	0.66140 (5)	0.0398 (5)	
C7	0.9615 (4)	−0.0520 (8)	0.62801 (7)	0.0342 (6)	
C8	1.1514 (4)	−0.2151 (8)	0.63137 (8)	0.0412 (7)	
H8	1.213 (5)	−0.241 (8)	0.6556 (8)	0.050 (9)*	
C9	1.2501 (5)	−0.3162 (9)	0.59971 (9)	0.0478 (8)	
H9	1.374 (5)	−0.431 (9)	0.6008 (9)	0.062 (10)*	
C10	1.1589 (5)	−0.2639 (9)	0.56450 (8)	0.0473 (8)	
C11	0.9690 (5)	−0.1103 (10)	0.56080 (8)	0.0514 (9)	
H11	0.902 (5)	−0.092 (9)	0.5362 (9)	0.063 (10)*	
C12	0.8682 (5)	−0.0049 (8)	0.59270 (8)	0.0417 (7)	
H12	0.742 (5)	0.108 (9)	0.5893 (8)	0.052 (9)*	
Cl13	1.28833 (18)	−0.3900 (3)	0.52474 (3)	0.0838 (4)	

### Atomic displacement parameters (Å^2^)

**Table:**

	*U*^11^	*U*^22^	*U*^33^	*U*^12^	*U*^13^	*U*^23^
N1	0.0321 (12)	0.0390 (16)	0.0327 (12)	−0.0020 (11)	0.0021 (10)	−0.0013 (11)
N2	0.0264 (11)	0.0380 (15)	0.0307 (11)	0.0052 (11)	−0.0004 (9)	−0.0004 (10)
C3	0.0266 (12)	0.0283 (15)	0.0352 (13)	−0.0003 (12)	0.0016 (10)	0.0043 (12)
O4	0.0316 (10)	0.0512 (14)	0.0445 (11)	0.0149 (10)	−0.0025 (8)	0.0003 (10)
C5	0.0307 (14)	0.0371 (17)	0.0342 (14)	0.0034 (13)	−0.0026 (11)	0.0025 (13)
O6	0.0404 (10)	0.0479 (13)	0.0307 (9)	0.0165 (10)	−0.0024 (8)	−0.0001 (9)
C7	0.0363 (14)	0.0361 (17)	0.0301 (13)	−0.0001 (13)	0.0013 (11)	−0.0013 (12)
C8	0.0369 (15)	0.049 (2)	0.0379 (15)	0.0075 (14)	−0.0049 (12)	−0.0007 (14)
C9	0.0384 (16)	0.051 (2)	0.0537 (18)	0.0085 (16)	0.0029 (14)	−0.0046 (16)
C10	0.0562 (19)	0.046 (2)	0.0406 (16)	0.0091 (16)	0.0109 (14)	−0.0036 (14)
C11	0.063 (2)	0.061 (2)	0.0305 (14)	0.0130 (19)	−0.0030 (14)	0.0010 (16)
C12	0.0418 (16)	0.045 (2)	0.0375 (15)	0.0105 (14)	−0.0040 (12)	0.0009 (13)
Cl13	0.0958 (8)	0.1032 (9)	0.0543 (5)	0.0323 (7)	0.0293 (5)	−0.0067 (6)

### Geometric parameters (Å, °)

**Table:**

N1—N2	1.420 (3)	C7—C12	1.381 (4)
N1—H1A	0.93 (4)	C7—C8	1.389 (4)
N1—H1B	0.89 (4)	C8—C9	1.367 (4)
N2—C3	1.319 (3)	C8—H8	0.94 (3)
N2—H2	0.86 (3)	C9—C10	1.376 (4)
C3—O4	1.237 (3)	C9—H9	0.92 (4)
C3—C5	1.499 (4)	C10—C11	1.372 (4)
C5—O6	1.419 (3)	C10—Cl13	1.734 (3)
C5—H5A	0.98 (3)	C11—C12	1.387 (4)
C5—H5B	0.94 (3)	C11—H11	0.96 (3)
O6—C7	1.372 (3)	C12—H12	0.93 (3)
			
N2—N1—H1A	107 (2)	O6—C7—C8	115.6 (2)
N2—N1—H1B	109 (2)	C12—C7—C8	119.8 (3)
H1A—N1—H1B	107 (3)	C9—C8—C7	120.1 (3)
C3—N2—N1	121.3 (2)	C9—C8—H8	121.4 (19)
C3—N2—H2	119.6 (19)	C7—C8—H8	118.4 (19)
N1—N2—H2	119.0 (19)	C8—C9—C10	120.0 (3)
O4—C3—N2	123.6 (2)	C8—C9—H9	123 (2)
O4—C3—C5	118.5 (2)	C10—C9—H9	117 (2)
N2—C3—C5	117.8 (2)	C11—C10—C9	120.5 (3)
O6—C5—C3	110.6 (2)	C11—C10—Cl13	120.3 (2)
O6—C5—H5A	111.3 (19)	C9—C10—Cl13	119.1 (3)
C3—C5—H5A	108.6 (17)	C10—C11—C12	120.0 (3)
O6—C5—H5B	108.7 (18)	C10—C11—H11	120 (2)
C3—C5—H5B	107.4 (19)	C12—C11—H11	120 (2)
H5A—C5—H5B	110 (3)	C7—C12—C11	119.5 (3)
C7—O6—C5	118.1 (2)	C7—C12—H12	122.2 (19)
O6—C7—C12	124.6 (3)	C11—C12—H12	118.1 (19)
			
N1—N2—C3—O4	−4.8 (4)	C7—C8—C9—C10	−1.6 (5)
N1—N2—C3—C5	173.5 (2)	C8—C9—C10—C11	0.1 (6)
O4—C3—C5—O6	−176.2 (3)	C8—C9—C10—Cl13	179.3 (3)
N2—C3—C5—O6	5.4 (4)	C9—C10—C11—C12	0.4 (6)
C3—C5—O6—C7	−164.9 (2)	Cl13—C10—C11—C12	−178.9 (3)
C5—O6—C7—C12	−6.7 (4)	O6—C7—C12—C11	178.9 (3)
C5—O6—C7—C8	174.5 (3)	C8—C7—C12—C11	−2.4 (5)
O6—C7—C8—C9	−178.4 (3)	C10—C11—C12—C7	0.8 (6)
C12—C7—C8—C9	2.8 (5)		

### Hydrogen-bond geometry (Å, °)

**Table:**

*D*—H···*A*	*D*—H	H···*A*	*D*···*A*	*D*—H···*A*
N1—H1A···O4^i^	0.93 (4)	2.51 (4)	3.160 (3)	127 (3)
N1—H1B···O4^ii^	0.89 (4)	2.15 (4)	3.020 (3)	165 (3)
N2—H2···N1^iii^	0.86 (3)	2.23 (3)	2.997 (3)	149 (3)
C8—H8···O4^iv^	0.94 (3)	2.51 (3)	3.376 (3)	153 (2)

Symmetry codes: (i) −*x*+1, *y*+1/2, −*z*+3/2; (ii) −*x*+1, *y*−1/2, −*z*+3/2; (iii) −*x*+2, *y*−1/2, −*z*+3/2; (iv) *x*+1, *y*−1, *z*.
